# Multiplex autoantibody screening in pediatric type 1 diabetes: POC Blot vs. ELISA for gluten-related disorders

**DOI:** 10.3389/fimmu.2025.1648451

**Published:** 2026-01-16

**Authors:** Adugna Negussie, Patricia Wusterhausen, Deselegn Muleta, Demise Mulugeta, Mesfin Bekele, Torsten Matthias

**Affiliations:** 1 Vaccine, Diagnostic and Medical Device Research and Development Directorate, Armauer Hansen Research Institute, Addis Adaba, Ethiopia; 2 Department of Medical Laboratory Sciences, College of Health Sciences and Medicine, Arsi University, Assela, Ethiopia; 3 Department of Research and Development, AESKU Diagnostics GmbH & Co. KG, Wendelsheim, Germany; 4 Adama Public Health Research and Referral Laboratory, Adama, Ethiopia; 5 AESKU Diagnostics GmbH & Co KG, Wendelsheim, Germany

**Keywords:** type 1 diabetes, autoantibody screening, gluten-related disorders, pediatric, point-of-care testing, ELISA, autoimmune disorders

## Abstract

**Background:**

Type 1 diabetes (T1D) is frequently associated with various autoimmune disorders, including gluten-related disorders (GRDs). Early screening for autoantibodies can provide insights into the co-occurrence of these conditions in pediatric patients. This study aims to evaluate the performance of a Point-of-Care Test (POC) BLOT based assay in comparison to the conventional enzyme-linked immunosorbent assay (ELISA) for multiplex autoantibody screening for gluten-related disorders in children with T1D.

**Methods:**

A cohort of pediatric and adolescent patients diagnosed with T1D was recruited for the study. Both POC-BLOT and ELISA methods were utilized to screen specific autoantibodies associated with GRDs, including anti-gliadin and anti-transglutaminase antibodies. Sensitivity, specificity, positive predictive value (PPV), and negative predictive value (NPV) were calculated for both screening methods. Statistical analyses were conducted to compare the results of the two assays.

**Results:**

A total of 192 participants were included in the study. The overall method agreement between the POC-BLOT and the ELISA test was approximately 90%. The PPV and NPV for both methods were also calculated indicating that the performance of the POC assay was comparable to ELISA. Additionally, results indicated that the POC assay provided faster results, highlighting its potential utility in clinical pediatric settings.

**Conclusion:**

The POC blot assay demonstrates promising potential as a reliable and rapid screening tool for autoantibodies in pediatric patients with T1D and gluten-related disorders in resource-limited settings. While further validation is needed, these findings suggest that POC testing could facilitate earlier diagnosis and improve the clinical management of coexisting autoimmune conditions in this population.

## Introduction

Celiac disease is a common autoimmune disorder characterized by an immune-mediated reaction to gluten in genetically predisposed individuals (1) and is classified as part of the spectrum of gluten-related disorders (GRDs). It frequently coexists with other autoimmune conditions, particularly type 1 diabetes mellitus (T1D). The prevalence of celiac disease among individuals with T1D is significantly higher than in the general population, ranging from 4% to 10%, depending on geographic and population studies (2). This association is likely driven by shared genetic factors, such as the HLA-DQ2 and HLA-DQ8 haplotypes, which predispose individuals to both conditions (2, 3). Understanding the relationship between these two diseases is crucial for early diagnosis and effective management.

The pathophysiology of both conditions involves a complex interplay of genetic, immunological, and environmental factors. In genetically susceptible individuals, the ingestion of gluten triggers an autoimmune response in the small intestine, leading to villous atrophy, malabsorption, and a broad spectrum of gastrointestinal and extraintestinal symptoms (4). Similarly, T1D results from an autoimmune attack on pancreatic beta cells, leading to insulin deficiency and dysregulation of glucose metabolism. The co-occurrence of these diseases suggests a shared autoimmune mechanism, with evidence pointing to the role of molecular mimicry, gut microbiota alterations, and immune dysregulation as potential contributing factors (5).

Clinically, the presence of celiac disease in T1D patients may complicate metabolic control and exacerbate symptoms such as unexplained hypoglycemia, growth retardation, delayed puberty, and nutritional deficiencies. Many patients with both conditions remain asymptomatic, making routine screening essential for early detection. Current guidelines recommend regular serological testing for celiac-specific autoantibodies (e.g., anti-tissue transglutaminase [tTG] and anti-deamidated gliadin peptide [DGP]) in newly diagnosed T1D patients and periodic follow-ups in those with negative initial results (6).

Despite the availability of sensitive and specific serological markers, diagnosing celiac disease in T1D patients can be challenging, particularly in cases of IgA deficiency, which is more prevalent in this population. In such instances, IgG-based serological tests or small intestinal biopsy remain essential for confirmation. Emerging diagnostic tools, such as multiplex autoantibody assays and POC screening methods, offer promising alternatives for early detection and risk stratification in pediatric populations (7).

A strict lifelong gluten-free diet (GFD) remains the only effective treatment for celiac disease. In T1D patients, adherence to a GFD has been associated with improved glycemic control, reduced gastrointestinal symptoms, and better overall quality of life. However, dietary compliance can be challenging, particularly in children and adolescents, necessitating multidisciplinary support from healthcare providers, including pediatric endocrinologists, gastroenterologists, and dietitians (8).

Given the high prevalence of celiac disease in T1D and its potential impact on metabolic and nutritional status, systematic screening and early intervention are crucial. Future research should focus on refining diagnostic strategies, understanding the immunopathogenic overlap between these conditions, and exploring novel therapeutic approaches to modulate the autoimmune response. This study aimed to compare the AESKUCARE^®^ Point-of-Care test (POC), a BLOT-based assay for near-patient testing, with the AESKULISA^®^ (an ELISA method) in serum samples of T1D in assessing biomarkers for celiac disease and GRDs in T1D Patients, contributing to a better understanding of diagnostics in pediatric endocrinology in resource-limited settings.

## Methods

This study analyzed archived serum samples collected from pediatric and adolescent patients (aged 1–18 years) with a confirmed diagnosis of T1D in Ethiopia. A total of 192 participants were included, of whom 121 (63%) were male. Participants were recruited from local hospitals, and inclusion criteria required a verified T1D diagnosis within the defined age range. The patients/participants or their legal guardians/next of kin provided written informed consent to participate in this study, and informed consent was obtained from legal guardians for all participants under 18 years of age. Ethical approval was granted by the relevant institutional review board.

### Sample collection and storage

Serum samples were collected following standard clinical protocols. Serum was separated and stored at –80 °C until testing to preserve biomarker stability and analytical integrity.

### Testing methods

POC testing was performed using the AESKUCARE^®^ Point-Of-Care test for determination of gluten associated specific IgA (human) (Aesku.Diagnostics GmbH & Co. KG, Germany, REF 860001). This membrane-based enzyme immunoassay is designed for the qualitative detection of IgA-class autoantibodies associated with gluten-related disorders. The assay enables multiplex detection of antibodies against a comprehensive antigen panel, including gliadin, DGP, tTG, tTG neo-epitopes (tTg neo), transglutaminase 3 (TG3), microbial transglutaminase neo-epitopes (mTG neo), microbial transglutaminase (mTG), Frazer’s Fraction and total IgA in human serum or plasma. Antigens are immobilized as discrete lines at defined positions on a nitrocellulose membrane to ensure spatial resolution and analytical specificity (**Table 1**).

This diagnostic tool plays a critical role in the evaluation of GRDs such as celiac disease and non-celiac gluten sensitivity, supporting early and accurate diagnosis. Its use is particularly valuable in the differential diagnosis of patients presenting with gastrointestinal and extraintestinal symptoms associated with gluten intolerance. However, it is important to note that antibody levels may be undetectable in individuals adhering to a gluten-free diet (9). In the POC immunoblot, total IgA was assessed simultaneously with the antigen-specific antibodies to identify possible IgA deficiency. A negative total IgA band indicated IgA deficiency, which may result in false-negative IgA-based serological findings.

In summary, AESKUCARE^®^ Gluten Related Disorders IgA represents a robust and precise assay for the serological assessment of gluten-related pathologies, contributing significantly to evidence-based clinical decision-making and patient management.

All serum samples were also tested using the AESKULISA^®^ CeliCheck New Generation (Aesku.Diagnostics GmbH & Co. KG, Germany, REF 3510), a solid-phase ELISA for the quantitative and qualitative detection of IgA and IgG antibodies targeting tTG neo-epitopes. This assay utilizes human recombinant tTG crosslinked with gliadin-specific peptides, thereby exposing clinically relevant neo-epitopes that enhance both sensitivity and specificity (10, 11). Standardized ELISA protocols were followed, including antigen-antibody incubation, washing steps, and detection via substrate-induced colorimetric changes. Optical density was measured spectrophotometrically, and quantitative results (**Table 1**) were analyzed using appropriate statistical software (**Table 2**).

**Table 1:** Possible assay results (qualitative evaluation) AESKUCARE^®^ Point-Of-Care test for determination of gluten associated specific IgA (human).

Test result	Antigen signal
Negative (Neg)	Signal intensity less than the cut-off calibrator (standard 1)
Equivocal (Equi)	Signal intensity approximately the same as the cut-off calibrator (standard 1)
Positive (Pos)	Signal intensity greater than the cut-off calibrator (standard 1)

**Table 2:** Interpretation of the results AESKULISA^®^ CeliCheck New Generation.

Normal range	Equivocal range	Positive results
< 12 U/ml	12–18 U/ml	>18 U/ml

### Data analysis

Data obtained from both testing methods were analyzed using IBM SPSS Statistics, version 29. Descriptive statistics were used to summarize the data, with continuous variables reported as means ± standard deviations and categorical variables expressed as frequencies (n) and percentages (%). Correlation and agreement between the POC and the ELISA method were assessed using appropriate statistical measures.

The overall method agreement between both testing methods was determined by comparing the qualitative outcomes of the POC and ELISA assays. A concordant (agreeing) result was defined when a patient tested positive in both the POC and the ELISA. If only one of the two methods yielded a positive result, this was considered a discordant (non-agreeing) outcome. The overall agreement rate was then calculated as the proportion of concordant results relative to the total number of tested samples.

Antigens that are not represented in the ELISA panel—specifically mTG, mTG neo, and TG3—were excluded from the calculation of overall agreement and Cohen’s kappa, as these targets are not detectable by the ELISA assay.

## Results

In the present study, 7 out of 192 participants (3.6%) exhibited low total immunoglobulin A (IgA) levels and negative antigen-specific responses, suggesting a potential selective IgA deficiency.

A total of 192 patient samples were initially analyzed. Two samples could not be evaluated by ELISA due to invalid measurements, and seven samples showed selective IgA deficiency in the immunoblot and were therefore excluded from the analysis. Consequently, 183 samples were included for the calculation of agreement between both assays.

The analysis demonstrated a substantial agreement between the ELISA and the immunoblot (κ = 0.64; 95% CI 0.51–0.77), with an overall concordance of 86.9%. The immunoblot showed a high sensitivity of 93.9%, correctly identifying nearly all ELISA-positive samples, and a specificity of 85.3%, accurately classifying the majority of ELISA-negative samples. The positive predictive value (58.5%) indicated that a portion of blot-positive results were not confirmed by ELISA, whereas the negative predictive value (98.5%) confirmed a very high reliability for negative findings (**Table 3**). Overall, the immunoblot exhibited strong diagnostic agreement with the ELISA, combined with slightly lower specificity.

**Table 3:** Diagnostic comparison between ELISA (reference assay) and POC assay.

Parameter	Result	Interpretation
*Sample size (n)*	183	—
*ELISA-positive samples*	33 (18.0%)	Reference positives
*ELISA-negative samples*	150 (82.0%)	Reference negatives
*POC-positive samples*	53 (29.0%)	—
*Overall agreement*	86.9%	Concordance between assays
*Cohen’s κ (weighted)*	0.64 (CI 95%: 0.51 – 0.77)	Substantial agreement
*Sensitivity*	93.9%	High detection of ELISA positives
*Specificity*	85.3%	Good discrimination of negatives
*Positive predictive value (PPV)*	58.5%	Moderate, due to false positives
*Negative predictive value (NPV)*	98.5%	Excellent reliability for negatives
*False-positive rate*	14.7%	POC positive/ELISA negative
*False-negative rate*	6.1%	POC negative/ELISA positive

**Table 4** summarizes the age-specific distribution of antibody positivity related to GRDs as detected by the POC immunoblot assay. Only POC-positive cases are included. Values represent the percentage of samples showing different numbers of positive antigen bands within each age group, relative to the total number of POC-positive cases per age category. Please note that the total number of patients varies between age groups.

**Table 4:** Age-stratified positivity rates (%) of gluten-related antibodies in children with type 1 diabetes.

Age (year)	n	mTG neo	TG3	tTG	tTG neo	mTG	Frazer’s Fraction	Gliadin	DGP
*2*	6	0.010	0.00	16.67	16.67	0.00	16.67	0.00	0.00
*3*	7	16.67	0.00	16.67	33.33	0.00	0.00	0.00	0.00
*4*	9	0.00	0.00	0.00	0.00	0.00	0.00	0.00	0.00
*5*	12	8.33	16.67	25.00	16.67	0.00	0.00	0.00	8.33
*6*	8	62.50	12.50	25.00	50.00	0.00	37.50	37.50	25.00
*7*	9	22.22	22.22	11.11	22.22	22.22	0.00	22.22	11.11
*8*	14	14.29	14.29	7.14	14.29	35.71	14.29	14.29	28.57
*9*	12	33.33	16.67	25.00	33.33	25.00	33.33	33.33	33.33
*10*	5	25.00	25.00	75.00	25.00	25.00	50.00	50.00	25.00
*11*	16	33.33	40.00	40.00	26.67	20.00	26.67	33.33	20.00
*12*	12	20.00	40.00	30.00	30.00	10.00	50.00	30.00	40.00
*13*	16	20.00	26.67	13.33	13.33	6.67	33.33	26.67	26.67
*14*	17	12.50	0.00	18.75	12.50	12.50	6.25	0.00	18.75
*15*	15	20.00	13.33	6.67	13.33	33.33	20.00	20.00	6.67
*16*	9	44.44	33.33	66.67	33.33	33.33	33.33	44.44	33.33
*17*	7	57.14	28.57	14.29	28.57	14.29	14.29	42.86	28.57
*18*	18	16.67	5.56	11.11	16.67	11.11	11.11	27.78	16.67

**Figure 1:**
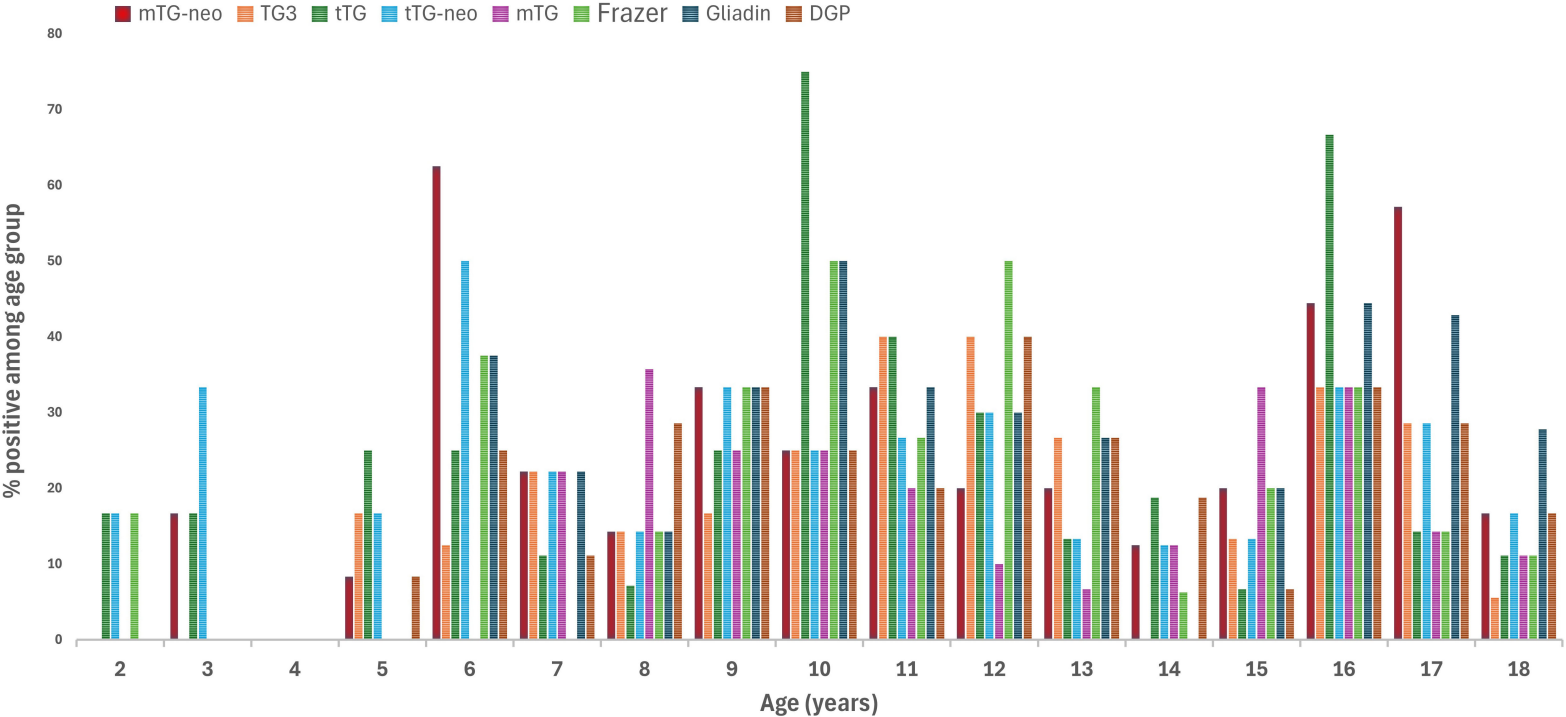
Age-dependent positivity rates of various gluten-related antibody markers in children with type 1 diabetes.

The bar graph (**Figure 1**) illustrates the percentage of antibody-positive cases across different age groups (2–18 years) for eight GRD serological markers: mTG-neo, TG3, tTG, tissue tTG-neo, mTG, Frazer’s Fraction, Gliadin, and DGP.

Each colored bar represents the proportion (%) of positive cases for the respective antigen within each age group. In children aged 2–8 years, a significant positive association was observed between age and the increase in gluten-related antigen positivity. Spearman’s rank correlation revealed a strong correlation (ρ = 0.79, p = 0.036) between age and the annual increase in antigen positivity (%). This indicates that within this early age range, each additional year of age was associated with a broader spectrum of antibody reactivity, reflecting a progressive expansion of gluten-related immune responses detectable by the multiplex point-of-care immunoblot.

## Discussion

### Clinical relevance and advantages of POC BLOT-based multiparametric testing in type 1 diabetes

The POC BLOT-based method demonstrated high concordance with conventional ELISA testing, with an overall concordance of 86.9%, confirming its reliability for rapid diagnostics in clinical settings. POC BLOT assays represent a significant advancement in autoimmune diagnostics, particularly for large-scale screening of conditions such as GRDs. Their ability to simultaneously detect multiple antibodies against various antigens—such as tTG, TG3, DGP, and related targets—enables a comprehensive immunological assessment from a single sample. This multiplex approach streamlines workflows, reduces turnaround time, and enhances diagnostic accuracy, even in resource-limited environments.

**Figure 2:**
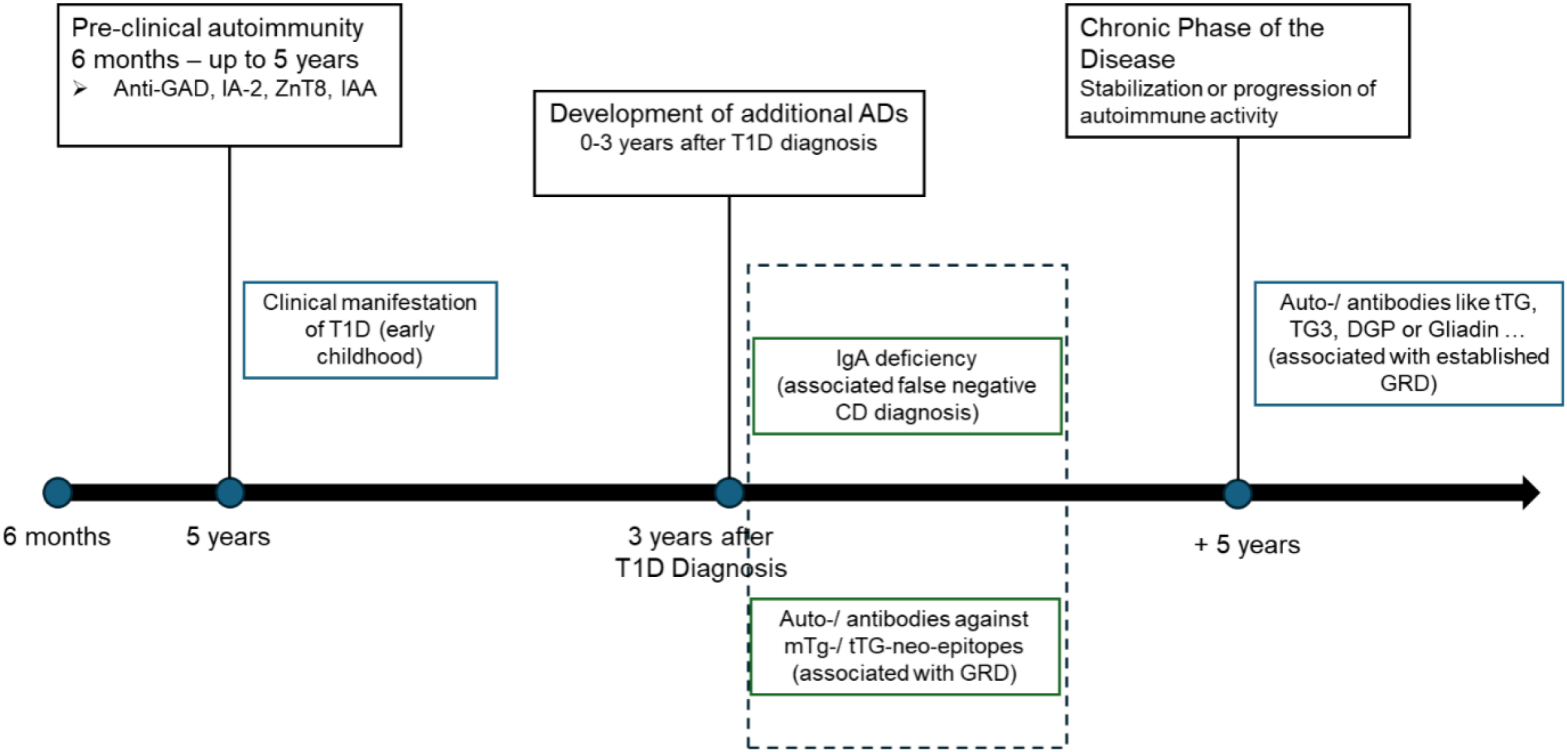
Stages of autoimmune progression in T1D and associated GRD autoantibody patterns. The schematic illustrates the temporal development of autoimmune activity in children with T1D. During the pre-clinical phase (approximately 6 months to 5 years), islet autoimmunity is initiated, typically characterized by the appearance of anti-GAD, IA-2A, ZnT8A, and IAA autoantibodies (14, 15). The clinical manifestation of T1D most frequently occurs in early childhood (16). Within 0–3 years after T1D diagnosis, additional autoimmune phenomena may develop, including IgA deficiency (associated with false-negative celiac disease results) and autoantibodies against microbial or tissue transglutaminase neo-epitopes (mTG/tTG-neo) related to gluten-associated immune activation (10, 11, 17). In the chronic phase, stabilization or progression of autoimmune activity occurs, with persistent TG3, tTG, DGP, or gliadin antibodies reflecting established gluten-related disorders (GRDs) (17–19).

The robustness of POC BLOT assays, including their resistance to matrix effects that can affect ELISA performance, supports consistent and reliable detection across diverse sample types (7, 12). Within the context of T1D, multiparametric antibody testing provides valuable insight into GRD and associated autoimmune responses. As illustrated in **Figure 1**, younger patients showed limited marker positivity, while older children exhibited increased frequencies and diversity of antibodies. This trend suggests a progressive broadening of the autoimmune response with age (13), potentially reflecting the evolution of gluten-related autoimmunity in T1D, shown in **Figure 2**.

Overall, integrating POC BLOT-based multiparametric testing into clinical practice offers substantial benefits for early detection and risk stratification in autoimmune diseases. By enabling comprehensive and rapid antibody profiling, this approach supports personalized management strategies and may ultimately improve patient outcomes.

### Antibodies against epidermal transglutaminase (TG3) and heir clinical relevance in type 1 diabetes and Dermatitis Herpetiformis

In this study, antibodies against TG3 were detected in 9.4% of individuals, highlighting their potential diagnostic relevance within the cohort. TG3 represents a key autoantigen in Dermatitis Herpetiformis (DH), a chronic, gluten-sensitive dermatosis characterized by pruritic, vesicular skin lesions. The presence of TG3 antibodies is considered a serological hallmark of DH and reflects the autoimmune response triggered by gluten exposure. Although not all patients with DH exhibit detectable TG3 antibodies, their identification provides valuable diagnostic information when interpreted alongside clinical and histopathological findings (20).

T1D and celiac disease share common genetic predispositions—particularly the HLA-DQ2 and HLA-DQ8 haplotypes—which contribute to an increased risk of celiac disease and related manifestations such as DH among T1D patients (20, 21). Notably, TG3 antibodies may emerge prior to the onset of clinical skin symptoms, suggesting their potential role as an early indicator of DH or subclinical celiac disease. The detection of TG3 antibodies in T1D patients could therefore support early identification of individuals at elevated risk, enabling timely diagnostic evaluation and management.

Integrating TG3 antibody testing into a broader serological assessment—alongside classical GRD antibodies—offers a more comprehensive understanding of GRD autoimmunity in T1D. Given that TG3 antibody production is influenced by gluten exposure, careful consideration of dietary factors is essential when interpreting results. Early recognition of TG3 positivity may help prevent complications associated with undiagnosed gluten-sensitive conditions, emphasizing the importance of serological screening in this high-risk population.

### Clinical implications of immunoglobulin A deficiency in type 1 diabetes

In the present study, 7 out of 192 participants (3.6%) exhibited low total IgA levels and negative antigen-specific responses, suggesting selective IgA deficiency. IgA deficiency is among the most common primary immunodeficiencies and is characterized by low or undetectable IgA levels in serum and secretions (22). This condition increases susceptibility to mucosal infections and is frequently associated with autoimmune diseases, including celiac disease and T1D. The relatively high prevalence observed in this pediatric cohort underscores the need for routine IgA screening in autoimmune populations.

IgA deficiency poses diagnostic challenges, particularly for celiac disease, as most standard assays—such as tTG-IgA and endomysium IgA (EMA-IgA)—rely on IgA-based immune responses and may yield false-negative results (1, 23, 24). In such cases, IgG-based markers should be used to ensure diagnostic accuracy (25). Rapid POC testing for total IgA provides immediate information on IgA status and facilitates appropriate follow-up testing.

T1D patients with IgA deficiency are at increased risk for other autoimmune conditions, such as celiac disease and autoimmune thyroid disorders (24). The absence of IgA compromises mucosal defense, promoting gastrointestinal inflammation and susceptibility to infection, which may exacerbate autoimmune manifestations (26). Early identification of IgA deficiency enables more accurate serological interpretation, supports timely dietary and therapeutic interventions, and improves long-term patient outcomes. Integrating IgA status assessment into routine T1D care is therefore essential for precise diagnosis and effective management of associated autoimmune disorders.

## Conclusion

This study highlights the promising utility of BLOT-based POC tests in detecting GRD related antigens in pediatric patients with T1D.

The ability of POC Blot assays to simultaneously detect a wide range of autoantibodies offers a comprehensive serological profile, thereby enhancing early diagnosis and individualized management of GRDs.

In particular, the detection of TG3 antibodies in T1D patients provides valuable insights into the risk of developing DH and other extraintestinal manifestations of GRDs. Additionally, the identification of patients with selective IgA deficiency underlines the need for tailored diagnostic algorithms that incorporate IgG-based serological testing to ensure accurate diagnosis and appropriate clinical management.

In addition to their diagnostic performance, POC assays offer potential cost advantages compared to conventional ELISA-based screening. Their reduced processing time, lower infrastructure requirements, and minimal need for specialized personnel may result in greater cost-effectiveness, particularly in resource-limited settings.

Overall, the findings support the integration of multiplex POC testing assays into routine screening protocols for autoimmune comorbidities in T1D. Their high diagnostic yield, rapid turnaround, and suitability for use in resource-limited settings position them as a valuable tool for improving outcomes through early intervention and personalized care strategies.

### Limitations of the study

This study has certain limitations. Detailed clinical information, such as the duration of T1D, the presence of other autoimmune comorbidities, and longitudinal follow-up data, were not available for the archived serum samples used. Consequently, potential associations between disease duration, comorbid autoimmune activity, and antibody prevalence could not be analyzed. Moreover, the study was based on cross-sectional data from a pediatric T1D cohort, which limits the ability to infer temporal dynamics of antibody development. These aspects should be addressed in future studies including comprehensive clinical metadata and prospective follow-up.

## Data Availability

The original contributions presented in the study are included in the article/supplementary material. Further inquiries can be directed to the corresponding author.
